# AdmixLD: fast genome-scale inference of ancestry disequilibrium in hybrid zones

**DOI:** 10.1093/bioinformatics/btag633

**Published:** 2026-08-27

**Authors:** Yannick Z Francioli, Richard H Adams, Kaas Ballard, Zachariah Gompert, Todd A Castoe

**Affiliations:** Department of Biology, University of Texas at Arlington, Arlington, TX, United States; Department of Entomology and Plant Pathology, University of Arkansas, Fayetteville, AR, United States; Department of Biology, University of Texas at Arlington, Arlington, TX, United States; Department of Biology, Utah State University, Logan, UT, United States; Department of Biology, University of Texas at Arlington, Arlington, TX, United States

## Abstract

**Summary:**

Hybrid zones represent powerful natural systems for studying reproductive isolation and speciation. One key genomic signature of genetic incompatibilities and epistatic interactions is linkage disequilibrium (LD)—non-random associations between loci from different lineage backgrounds, generated by selection against maladaptive allele combinations. However, admixture alone induces strong genome-wide LD in hybrid populations, obscuring selection-driven signals. Here, we present AdmixLD, a fast, scalable C++ tool for genome-wide LD scanning in hybrid zones that estimates LD using partial correlation to control for individual hybrid index. By removing admixture-driven covariance, AdmixLD enhances detection of locus-specific associations and enables genome-scale identification of candidate barrier loci and interacting genomic regions.

**Availability:**

The software and its code source are available at https://github.com/yzfranci/AdmixLD, and scripts for the data analysis are available at https://github.com/yzfranci/AdmixLDAnalysis.

## 1 Introduction

Natural hybrid zones between divergent populations provide valuable systems to study mechanisms of reproductive isolation and speciation. Hybrid zones, particularly those formed via secondary contact between previously isolated populations, have long been described as natural laboratories in which the consequences of gene flow, selection, and genetic incompatibilities can be observed directly (Barton and Hewitt 1985, Hewitt 1988, Harrison 1993, Abbott *et al.* 2013). When divergent genomes come into contact, hybridization disrupts coadapted gene complexes, separating alleles that evolved in different genetic backgrounds and generating incompatibilities that reduce hybrid fitness. Multi-locus interactions are therefore expected to play a central role in the evolution and maintenance of reproductive isolation between diverging lineages. A key genomic signature of such incompatibilities is the generation of non-random associations among loci—linkage disequilibrium (LD)—as selection acts against maladaptive allele combinations in hybrid individuals (Szymura and Barton 1991, Nürnberger *et al.* 1995, Payseur 2010, Pool 2015). Such incompatibilities are recognized as pervasive features of natural hybrid zones, and their polygenic nature is consistent with reproductive isolation being driven by interactions across many loci (Arnegard *et al.* 2014, Schumer *et al.* 2014, Christie and Strauss 2018, Nikolakis *et al.* 2022).

When ongoing gene flow from parental populations generates spatially structured ancestry across a transect, mixture LD (Falush *et al.* 2003) arises purely from covariance in shared ancestry proportions across individuals, independent of any selection against incompatible combinations (Barton and Hewitt 1985, Pfaff *et al.* 2001). This represents implications for sampling itself: because mixture LD scales with variation in admixture proportions across sampled individuals, overrepresentation of parental-type individuals from zone margins will further inflate inter-locus correlations. Admixture LD arises from a related but distinct process: in recently admixed genomes, recombination has not yet had time to break down long ancestry tracts inherited from parental populations, generating correlations among physically distant loci that reflect shared ancestry tracts rather than selection (Falush *et al.* 2003). Together, mixture LD and admixture LD form a pervasive background of ancestry-driven LD in hybrid populations that is unrelated to selection. LD in excess of this background is exceptionally informative for identifying barrier loci and genomic regions that impede gene flow, and for dissecting the genetic architecture underlying reproductive isolation (Gavrilets 2004, Nosil *et al.* 2009, Flaxman *et al.* 2014, Lindtke and Buerkle 2015, Ravinet *et al.* 2017).

Accounting for individual genome-wide admixture and focusing on the correlation between residuals—excess ancestry LD—is therefore crucial to distinguish non-random associations among loci driven by selection from those generated by mixture LD alone, particularly in heavily structured systems such as hybrid zone transects. The statistical foundations for estimating LD in structured populations have been established by several key methodological contributions (Mackay and Powell 2007, Lin *et al.* 2012, Mangin *et al.* 2012), and Schumer and Brandvain (Schumer and Brandvain 2016) extended this framework explicitly to hybrid zones, providing a theoretical basis for separating mixture-driven from selection-driven associations in admixed genomes. Despite these advances, a practical gap remains: while fast, genome-scale tools for estimating LD in structured populations exist (Bercovich *et al.* 2025), they are designed for LD pruning rather than the detection of outlier loci, and are not tailored specifically for hybrid zone analyses. To address this, we present AdmixLD: a dedicated, scalable C++ software for genome-wide intra- and interchromosomal excess ancestry LD scans in hybrid zones that explicitly accounts for individual admixture proportions, facilitating the identification of selection-driven LD.

## 2 Methods

AdmixLD is implemented in C++ (v. 17). The tool accepts local ancestry tracts or SNPs as ancestry markers encoded in standard VCF/BCF files through HTSlib (Bonfield *et al*. 2021), as well as standard most likely sub-population (MSP) files from RFMIX (Maples *et al*. 2013). AdmixLD first computes a per-sample hybrid index (HI)—the genome-wide proportion of ancestry. When local ancestry tracts are provided, HI is estimated using a tract-length-weighted estimator: HI_i_ = Σ_j_ d_ij_ · L_j_/(2 · Σ_j_ L_j_), where d_ij_ is the ancestry dosage (0 or 1 for phased haplotypes, 0, 1, or 2 for diploids) for individual i at tract j and L_j_ is the inferred tract length. When a VCF encodes SNPs rather than ancestry tracts, two options are available. If the markers are assumed ancestry-diagnostic (parental allele frequencies p_1_ ≈ 1, p_2_ ≈ 0), a non-weighted HI can be computed directly from dosages. Alternatively, if the variants are not fixed between the two parental populations, an allele frequencies file can be supplied. In this case, each marker is first polarized so that the ancestry-1 allele is the higher-frequency allele in parental population 1 (p_1_ ≥ p_2_); for any marker where p_2_ > p_1_ the dosage is flipped, d′_ij_ = dmax − d_ij_ (with p_1_ and p_2_ swapped accordingly), where dmax = 2 for diploid and 1 for phased haplotype data. HI is then estimated using a δ²-weighted method-of-moments estimator (Buerkle 2005): HI_i_ = [Σ_j_ (x_ij_/2 − p_2j_) · δ_j_]/Σ_j_ δ_j_² where δ_j_ = p_1j_ − p_2j_ is the allele frequency difference between parental populations at marker j. A leave-focus-chromosomes-out HI computation mode is also available to avoid confounding the focal chromosome’s signal with its own contribution to the global HI estimate (Schumer and Brandvain 2016). Alternatively, a file specifying hybrid indices can be provided directly by the user.

Each marker’s dosage vector is residualized against the genome-wide ancestry and *z*-scored, isolating locus-specific ancestry variation from the background admixture signal common to all loci. For the jth marker with dosage vector d_j_ across n individuals, residuals are computed using the expected dosage under Hardy–Weinberg equilibrium in an admixed population. When parental allele frequencies are not provided (markers assumed ancestry-diagnostic, p_1_ ≈ 1, p_2_ ≈ 0), residuals are computed as:


$$\varepsilon_{\text{ij}}=d_{\text{ij}}-{\;2\text{HI}}_{i}\left(\text{diploid}\right)\;\varepsilon_{\text{ij}}=d_{\text{ij}}-{\;\text{HI}}_{i}\left(\text{phased}\right)$$

When parental allele frequencies are provided, residuals incorporate the marker-specific allele-frequency contrast δ_j_ = p_1j_ − p_2j_ (≥ 0 after polarization):


$$\varepsilon_{\text{ij}}=d_{\text{ij}}-2\left(p_{2j}+\delta_{j}\cdot {\;\text{HI}}_{i}\right)\;\left(\text{diploid}\right)\;\varepsilon_{\text{ij}}=d_{\text{ij}}-\left(p_{2j}+\delta_{j}\cdot {\;\text{HI}}_{i}\right)\;\left(\text{phased}\right)$$

In both cases the residuals are mean-centered and standardized across samples, Z_ij_ = (ε_ij_ − mean(ε_j_))/SD(ε_j_)—where mean(ε_j_) and SD(ε_j_) are computed across samples i for each marker j—and markers with zero residual standard deviation are excluded as uninformative. Excess ancestry LD is then quantified as the Pearson correlation between all pairwise standardized residual vectors.

AdmixLD supports both interchromosomal pairs (the default) and intrachromosomal pairs, the latter optionally filtered for maximum genomic distance threshold. To avoid computing and storing correlations across all marker pairs—which can produce extremely large output files at genome scale—AdmixLD estimates the empirical distribution of partial correlation values using reservoir sampling (default capacity: 2,00,000 values), which provides a memory-efficient approximation of the full distribution. This empirical distribution can then be used to inform the choice of an outlier detection threshold.

Additionally, we implemented a false discovery rate (FDR) framework based on an empirical null distribution (Efron 2004, 2012), which assumes that most tested pairs are neutral and only a minority reflect true signal. We calibrate a separate null distribution for each chromosome-pair block (interchromosomal) or distance bin (intrachromosomal). Within a block, all pairwise correlations are Fisher z-transformed (*z* = atanh(r)) and modeled as Gaussian, N(μ_0_, σ_0_²), with μ_0_ = median(*z*) and σ_0_ = 1.4826 × median(|*z* − median(*z*)|), a robust estimator of the null distribution’s spread that limits the influence of true signal in the tails. Each z-score is then recalibrated against its block-specific null distribution (z* = (*z*  −  μ_0_)/σ_0_) and converted to a one-sided *P*-value per tail, since the empirical null distribution is not guaranteed to be symmetric about zero. We then apply Storey’s *q*-value procedure (Storey 2002, Storey and Tibshirani 2003) per tail to control the FDR at a user-specified threshold. To bound memory use, null distribution calibration draws on a reservoir sample of *z*-scores per block (200 000 by default) rather than the full pairwise set. For intrachromosomal scans, a minimal distance can be set for marker pairs and the null distribution calibration is done separately within log-spaced distance bins, as physical linkage inflates LD independent of admixture. The same framework was applied on traditional LD output using a custom Python script.

We demonstrated the application of AdmixLD using simulated hybrid zone data, which were generated using SLiM version 5.2 (Haller and Messer 2017). Each simulation modelled a 30-deme stepping-stone hybrid zone evolving under a Wright-Fisher process, with deme sizes of 100 individuals and an inter-deme migration rate of 0.1 per generation. Individual genomes consisted of three one-Morgan chromosomes, with 1000 fixed ancestry markers distributed at equal intervals, and simulations were run for 500 generations. For each replicate, two VCF files of ancestry markers were output to evaluate the effect of sampling scheme: one containing 200 randomly sampled hybrids (balanced sampling), and one with unbalanced sampling comprising 50 individuals with hybrid index (HI) < 0.05, 100 with 0.05 ≤ HI ≤ 0.95, and 50 with HI > 0.95.

We simulated several incompatibility architectures. First, a neutral simulation without any selected loci. We then modelled classical two-locus Dobzhansky–Muller incompatibility scenarios (DMI) involving different numbers of loci pairs (1, 2, 3 and 6) with individual fitness defined as w = 1 − s × |d_1_ − d_2_|/2, where d_1_ and d_2_ are ancestry dosages at each paired locus (0, 1, or 2 alleles derived from population 2; loci are fully differentiated between parental populations) and *s* = 0.5. We also simulated a single-DMI scenario under weaker selection (*s* = 0.05). We modeled coadaptation incompatibility involving six fully differentiated loci (two per chromosome), where fitness was penalized by variance in dosage across loci: *w* = 1 − *s* × Var(*d*), where d is the vector of per-locus dosages and *s* = 0.5. Finally, to test the effect of non-fixed allele-frequency variance between populations, we repeated the six-locus coadaptation scenario, but with 1000 markers spanning a range of allele frequencies. Each simulation type was run for 100 replicates.

Excess Ancestry LD was computed with AdmixLD on VCF files from SLiM, and benchmarked against traditional LD estimated with vcftools ‑‑interchrom-geno-r2 (Danecek *et al.* 2011). Although vcftools is not designed to account for admixture structure, it provides a baseline for illustrating the magnitude of bias introduced by uncorrected ancestry covariance. For each simulation, the coefficient of determination (*R*^2^) distribution was approximated by randomly sampling 200 000 marker pairs. To estimate overall *R*^2^ distributions in each simulation replicate, we used 200 000 randomly sampled marker pair *R*^2^ values. The resulting background distributions were then used to compute *z*-scores for selected loci *R*² for every simulation replicate, quantifying their deviation from the genomic background. Distributions for visualization purposes (violin plots) were obtained by pooling 200 000 randomly sampled marker pair *R*^2^ values across five replicates per simulation type.

To assess signal enrichment at selected loci, we computed the median of the pooled true selected locus pair *R*^2^ distribution across simulation replicates, along with their background *z*-scores (Supplemental Table S2, available as supplementary data at *Bioinformatics* online), and the 5th percentile to provide a conservative lower-bound estimate. Sensitivity and specificity of the FDR-based outlier detection were assessed using the true positive rate as the number of true hits among selected pairs divided by the total number of selected pairs across all replicates, and the false positive rate as the number of false hits divided by the total number of non-selected loci pairs, excluding pairs where both markers fall within 2 cM of a selected locus to avoid confounding effects from indirect selection. Additionally, we computed a region hit rate: the proportion of marker pairs with both markers within 2 cM of a selected locus that were called significant, used to quantify enrichment of hits near true loci relative to the background rate.

We also demonstrated an empirical application of AdmixLD and compared its performance against traditional LD estimation using published ancestry track data from a rattlesnake hybrid zone (Francioli *et al.* 2025). Both AdmixLD and vcftools ‑‑interchrom-geno-r2/ ‑‑geno-r2 were applied to compute Interchromosomal excess ancestry LD between local ancestry tracts of chromosomes 6 and 7, while intrachromosomal excess ancestry LD was computed for chromosome 3.

## 3 Results

**Figure 1:**
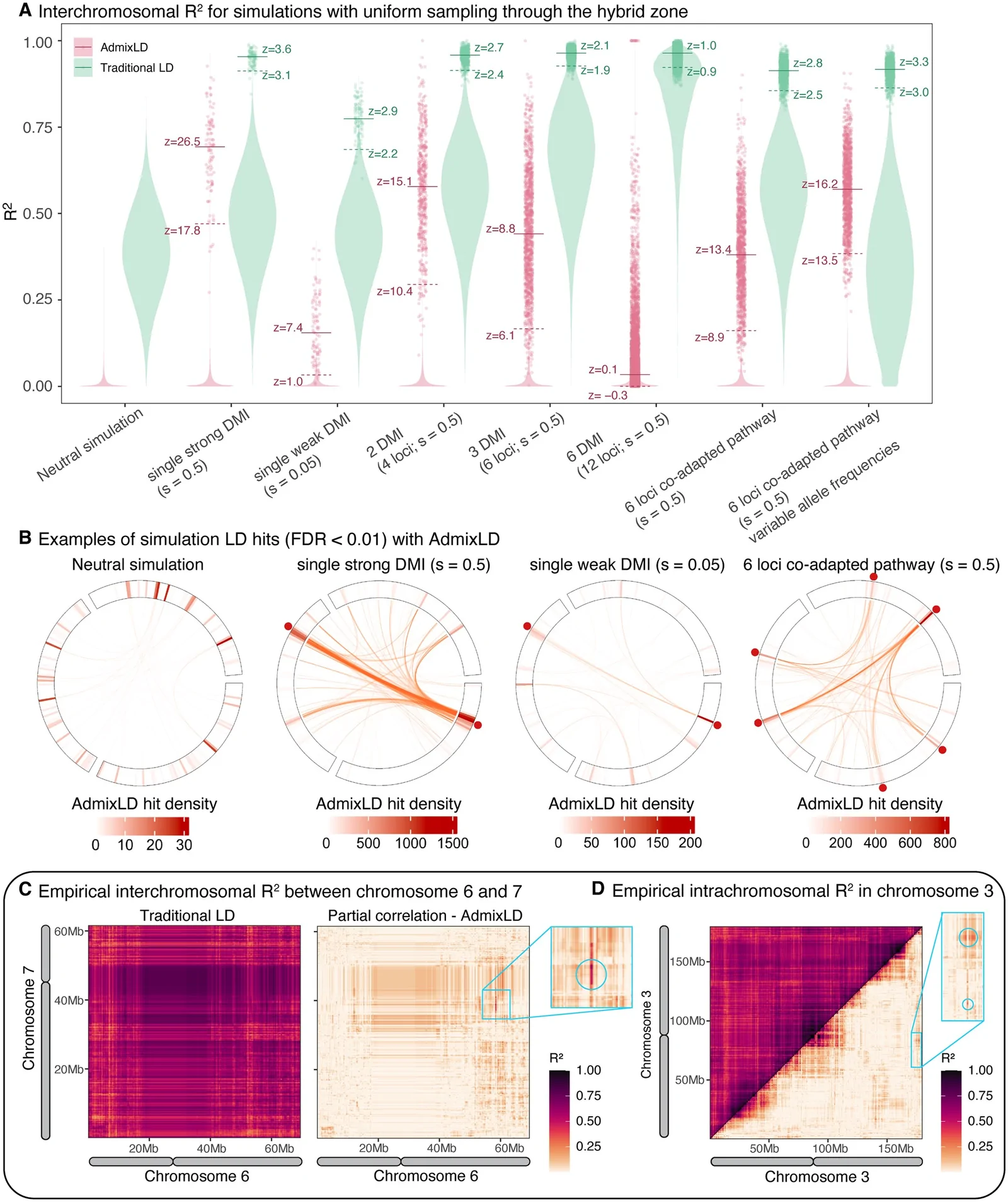
Comparison of excess ancestry LD (partial correlation) and traditional LD across different simulation scenarios, and on empirical data. (A) Overall *R*^2^ distributions compared to selected loci pairs. Shaded distributions show background *R*^2^ values across all locus pairs; colored points indicate *R*^2^ values for incompatible locus pairs. Pink: excess ancestry LD (AdmixLD); green: traditional LD. Dashed and solid horizontal lines indicate the 5th percentile and median *z*-scores of incompatible locus pairs, respectively. (B) Structure of identified outlier pairs (using AdmixLD implemented FDR method) across simulated genomes, shown for single simulation replicates. Orange lines represent LD between loci pairs identified as outliers (FDR < 0.01), red dots show the location of loci under selection, and the outer track show the density of outlier hit (see Figure S2, available as supplementary data at *Bioinformatics* online for all simulation scenarios). (C) Empirical interchromosomal and intrachromosomal LD. between chromosomes 6 and 7 in a rattlesnake hybrid zone, estimated using excess ancestry LD (right) and traditional LD (left). (D) Empirical intrachromosomal for chromosome 3 of the same hybrid zone, displayed as a triangular heatmap with excess ancestry LD in the upper-left triangle and traditional LD in the lower-right triangle. Blue rectangles zoom in regions with identified outliers, and blue circles show the location of specific outlier region pairs (FDR < 0.01 for panel C and FDR < 0.05 for panel D).

Across all simulated scenarios, AdmixLD’s background *R*² remained low and stable, ranging from 0.0126 (neutral) to 0.0275 (six-pair multi-DMI; Fig. 1A, Supplemental Table S1, available as supplementary data at *Bioinformatics* online). By contrast, traditional LD’s background R² scaled with the extent of genome-wide selection, from 0.399 (neutral) to 0.836 (six-pair multi-DMI). These results highlight how the signal of admixture-driven and selection-driven associations are entangled when using traditional LD. In the single-DMI scenario with strong selection (*s* = 0.5), selected loci pairs showed extreme *R*² enrichment relative to background, expressed as a *z*-score (deviation from the background *R*² distribution) of 26.53, far exceeding traditional LD (*z* = 3.55). As expected, reducing selection strength lowered enrichment: at *s* = 0.05, AdmixLD’s median *z*-score dropped to 7.40, compared to 2.91 for traditional LD (Fig. 1A, Supplemental Table S1, available as supplementary data at *Bioinformatics* online).

Across all simulations, AdmixLD’s *z*-scores for selected loci pairs remained well above traditional LD’s, with one exception: the six-pair multi-DMI scenario under extreme genome-wide selection (Fig. 1A, Supplemental Table S1, available as supplementary data at *Bioinformatics* online). Enrichment declined as more independent DMI pairs were placed under selection: median *z*-scores fell from 26.53 (single strong DMI) to 15.11 (two pairs) to 8.81 (three pairs). This decline coincided with a rise in AdmixLD’s own background *R*² (0.0149–0.0275), consistent with genome-wide selection increasingly affecting the background against which true selected loci pairs are compared. In the six-pair multi-DMI scenario, AdmixLD’s median *z*-score dropped to 0.09 (5th percentile *z* = −0.275; vs. 1.04 and 0.931 for traditional LD), indicating that at this level of genome-wide selection, background and true-pair *R*² distributions were no longer distinguishable. Even here, however, a subset of selected pairs still showed extreme *R*² values under AdmixLD—highlighting a distinction that was obscured under traditional LD due to its far more inflated background *R*² (0.83; Fig. 1A). Notably, while detection power decreases as genome-wide selection increases (i.e., in the three-pair multi-DMI scenario above), the number of loci alone does not erode the signal: the pathway scenario, which involves six selected loci but penalizes fitness through variance in dosage (maximum s = 0.5), retained stronger enrichment (median *z* = 13.4, 5th percentile *z* = 8.85). The scenario with variable allele frequency markers showed an increase in the median *z*-score for both AdmixLD (13.4–16.2) and traditional LD (2.80–3.28).

Under unbalanced sampling, traditional LD’s background *R*² increased substantially across all scenarios (e.g. 0.399–0.567 in the neutral simulation; Fig.1A, Supplemental Fig. S1, Supplemental Table S1, available as supplementary data at *Bioinformatics* online), while AdmixLD’s background *R*² rose only marginally (0.0126–0.0162). This asymmetry across methods carried through to enrichment: AdmixLD’s *z*-scores at selected loci pairs dropped only slightly relative to balanced sampling (e.g. 26.53–23.65 in single strong DMI) and remained far above that of traditional LD, which declined further under imbalance (e.g. 3.55–2.83).

At FDR < 0.01, the FDR-based outlier detection showed low false positive rates across all scenarios, with background hit rates ranging from 0.00008 (neutral) to 0.0057 (six-pair multi-DMI), broadly mirroring the slight increase in background *R*² with architecture complexity (Supplemental Table S2, available as supplementary data at *Bioinformatics* online). True positive rates (TPR) closely tracked the enrichment patterns described above: TPR was 1.0 for single-pair strong-DMI and two-pair multi-DMI, declined to 0.93 at three pairs, and collapsed to 0.12 at six pairs. In multi-DMI scenarios, cross pair hit rates—false positives among pairs combining loci from different true DMI pairs—were high and close to TPR, likely reflecting a coupling effect between DMI, and highlighting the challenge of separating distinct barrier loci. Detection power was sensitive to selection strength, as TPR dropped to 0.3 under weaker selection (*s* = 0.05). In contrast, both pathway architectures retained high TPR (0.89 with fixed markers, 0.998 with variable allele frequencies) despite also involving six loci, indicating that detection power depends on how selection is distributed among loci rather than based on selected locus count alone. AdmixLD outlier density concentrates specifically around selected loci (within 0.2cM: 10–1059× enriched over background, lowest at six-pair multi-DMI and highest for single-strong-DMI; Fig 1B, Supplemental Fig. S2, Supplemental Table S2, available as supplementary data at *Bioinformatics* online), scaling with selection strength and structure, in contrast with the low-density background under neutrality. Notably, the enrichment dropped in the pathway case with variable allele frequency, likely due to lower correlation between the selected loci and surrounding loci, as well as a higher false positive rate. Applying the same FDR framework to traditional LD yielded no outliers in any scenario, a direct consequence of its inflated background *R*², which prevents selected loci pairs from being statistically distinguished from the background distribution regardless of true enrichment.

AdmixLD took 3 minutes and 25 seconds single-threaded (Intel Core i7-3770 3.40 GHz) with the default tile size (1024 columns per tile; see Methods) to compute partial correlations between loci of the empirical dataset, including 580 million ancestry tracts pairs, demonstrating efficient genome-scale computation.

In our empirical application, interchromosomal excess ancestry LD computed with AdmixLD revealed a prominent region of elevated *R*² between chromosomes 6 and 7, against a near-zero background (Fig. 1C), highlighting strong localized signals. Using the implemented FDR framework, this region pair (∼58 Mb on chromosome 6 and ∼ 39 Mb on chromosome 7) was the only significant outlier at FDR < 0.01 for this chromosome pair. Traditional LD estimated with vcftools, by contrast, showed broadly elevated *R*² across nearly the entire chromosome pair, obscuring discrete signals (Fig. 1C). Intrachromosomal excess ancestry LD (showed on chromosome 3; Fig. 1D) exhibited a clear distance-decay pattern, with *R*² highest among physically proximate loci and declining sharply with genomic distance, consistent with recombination breaking down ancestry associations. This pattern was largely absent in traditional LD estimates due to a uniformly elevated background. While no outliers were found at FDR < 0.01 on chromosome 3, two region pairs were significant at FDR < 0.05: ∼175 Mb with ∼84 Mb, and ∼175 Mb with ∼66 Mb (Fig. 1D).

## 4 Discussion

AdmixLD provides a computationally efficient approach to detect excess ancestry LD in hybrid zones, where admixture alone generates strong LD signals that challenge traditional methods (Barton and Hewitt 1985, Pfaff *et al.* 2001). By explicitly accounting for genome-wide admixture and using partial correlation (Lin *et al.* 2012, Schumer and Brandvain 2016), AdmixLD isolates LD in excess of the mixture LD background, enriching the signal for selection-driven LD regions and facilitating reliable identification of outlier locus pairs and genomic regions. AdmixLD also implements an outlier detection method based on an empirical null distribution (Efron 2004, 2012) and Storey’s q-value FDR procedure (Storey 2002, Storey and Tibshirani 2003), which our simulations show performs well across a range of incompatibility architectures with low false positive rates. This built-in framework is intended as a convenient default rather than the only valid approach, and AdmixLD’s outputs are provided in standard formats, allowing users to apply alternative FDR or outlier-detection methods.

AdmixLD combined with this outlier detection method performed well across simulation scenarios (strong and weak DMIs, multi-locus coadapted pathways), achieving high true positive rates while keeping background false positive rates very low. It should be noted, however, that given the large number of background comparisons genome-wide, the absolute number of false positives can still be substantial even at a low per-comparison rate. Structural variation, alleles under local adaptation and assortative mating can also generate confounding LD signals unrelated to epistasis. Accordingly, AdmixLD’s outlier detection would ideally be combined with complementary evidence. Enrichment of outlier density in the vicinity of candidate incompatibility loci, consistency of LD outliers across independent hybrid zone transects of the same species pair, and alternative statistics such as the decay of heterozygosity variance (Li *et al.* 2022) could be used to corroborate inferences. Finally, strong genome-wide selection can itself elevate background LD (Feder *et al.* 2014), as selection acting across many loci simultaneously increasingly perturbs the ancestry structure against which any single locus pair is compared, reducing the method’s power to distinguish true incompatibilities from background.

Controlling for mixture LD confers robustness under imbalanced sampling, which is crucial since hybrid zones are rarely sampled in a uniform way with equal representation across hybrid indices. By using tiled matrix multiplication, combined with OpenMP parallelization across chromosome pairs—or chromosomes for intrachromosomal inferences—AdmixLD provides efficient computation, enabling genome-scale analyses of large marker sets.

In conclusion, AdmixLD fills an important methodological gap in hybridization and reproductive isolation research, which increasingly relies on whole genome datasets (Payseur and Rieseberg 2016, Moran *et al.* 2021). By explicitly correcting for genome-wide admixture, AdmixLD increases power to detect selection-driven excess ancestry LD compared to traditional LD measures, enabling more accurate identification of the genetic architecture of interacting loci and genomic regions under selection in hybrid zones.

## Supplementary Material

btag633_Supplementary_Data

## Data Availability

AdmixLD is available for download at https://github.com/yzfranci/AdmixLD, and it can be installed through bioconda (https://anaconda.org/bioconda/admixld). AdmixLD is also archived on Zenodo (https://doi.org/10.5281/zenodo.21298533). The scripts for the figures and analysis are available at https://github.com/yzfranci/AdmixLDanalysis. The empirical data used is available on Dryad at https://doi.org/10.5061/dryad.kh18932k9.
