# Supplementary material for: AdmixLD: fast genome-scale inference of ancestry disequilibrium in hybrid zones
Source: Bioinformatics. 2026 Aug 27;42(9):btag633. doi: 10.1093/bioinformatics/btag633 (PMC13533569; doi:10.1093/bioinformatics/btag633)
Supplement: Supplementary file 1 [file btag633_supplementary_data.pdf]

Supplementary Information (Francioli et al.)

Including:

Supplementary Tables S1-S2

Supplementary Figures S1-S2

Supplementary Table S1. Enrichment of R^2 at selected loci relative to the genome-wide background, by selection scenario, tool (ADmixLD vs. Traditional LD), and sampling scheme (balanced/unbalanced). Median R^2 and R^2 5th percentile are the median and 5th percentile of exact selected-locus pairs' R^2. Z-score median and z-score 5th percentile are the median and 5th percentile of exact selected-locus pairs' z-scores (measured within each replicate, the selected pair(s)'s R^2 is compared against the simulation replicate's own background R^2 mean and SD). Background mean R^2 and background SD R^2 are the mean and SD of all marker-pairs's R^2 pooled across all simulation replicates.

Selection type	Tool	Sampling	Background mean R^2	Background SD R^2	Median R^2	z-score median	R^2 5th percentile	z-score 5th percentile
neutral	AdmixLD	balanced	0.0126	0.0187	NA	NA	NA	NA
neutral	Traditional LD	balanced	0.3993	0.1243	NA	NA	NA	NA
single DMI (strong)	AdmixLD	balanced	0.0149	0.025	0.6928	26.5357	0.4703	17.8061
single DMI (strong)	Traditional LD	balanced	0.4764	0.1334	0.9537	3.5521	0.9125	3.1351
single DMI (weak)	AdmixLD	balanced	0.0128	0.019	0.155	7.4051	0.033	0.988
single DMI (weak)	Traditional LD	balanced	0.415	0.1251	0.7742	2.909	0.6852	2.1855
multi-DMI (2 loci pairs)	AdmixLD	balanced	0.0193	0.0358	0.5779	15.1181	0.2945	10.444
multi-DMI (2 loci pairs)	Traditional LD	balanced	0.5784	0.1391	0.9583	2.7042	0.9142	2.3512
multi-DMI (3 loci pairs)	AdmixLD	balanced	0.0243	0.0468	0.4412	8.8176	0.1664	6.1346
multi-DMI (3 loci pairs)	Traditional LD	balanced	0.6669	0.1404	0.9641	2.1093	0.9268	1.8952
multi-DMI (6 loci pairs)	AdmixLD	balanced	0.0275	0.0463	0.0336	0.0908	0.0003	-0.2752
multi-DMI (6 loci pairs)	Traditional LD	balanced	0.8361	0.1214	0.9642	1.0396	0.9228	0.9311
Pathway (6 loci)	AdmixLD	balanced	0.0161	0.0271	0.3806	13.4176	0.1613	8.8541
Pathway (6 loci)	Traditional LD	balanced	0.5302	0.1368	0.9134	2.8064	0.8556	2.508
Pathway (6 loci; var. allele. freq.)	AdmixLD	balanced	0.02	0.0333	0.57	16.2077	0.3838	13.4955
Pathway (6 loci; var. allele. freq.)	Traditional LD	balanced	0.3477	0.1724	0.917	3.2845	0.8634	2.9849
neutral	AdmixLD	unbalanced	0.0162	0.0242	NA	NA	NA	NA
neutral	Traditional LD	unbalanced	0.5673	0.1319	NA	NA	NA	NA
single DMI (strong)	AdmixLD	unbalanced	0.0175	0.0285	0.685	23.6524	0.5065	17.1597
single DMI (strong)	Traditional LD	unbalanced	0.5985	0.13	0.9651	2.8342	0.9402	2.5155
single DMI (weak)	AdmixLD	unbalanced	0.0161	0.0242	0.1897	7.1737	0.0341	0.8011
single DMI (weak)	Traditional LD	unbalanced	0.5709	0.1302	0.8744	2.3118	0.7998	1.7816
multi-DMI (2 loci pairs)	AdmixLD	unbalanced	0.0207	0.038	0.5888	14.8494	0.2793	9.3476
multi-DMI (2 loci pairs)	Traditional LD	unbalanced	0.6416	0.1317	0.9648	2.4537	0.9292	2.1562
multi-DMI (3 loci pairs)	AdmixLD	unbalanced	0.0247	0.0474	0.4297	8.747	0.1636	5.8041
multi-DMI (3 loci pairs)	Traditional LD	unbalanced	0.6877	0.1359	0.9647	2.0466	0.9344	1.8198
multi-DMI (6 loci pairs)	AdmixLD	unbalanced	0.0191	0.0252	0.0125	-0.2603	0.0001	-0.5469
multi-DMI (6 loci pairs)	Traditional LD	unbalanced	0.7395	0.135	0.9023	1.164	0.8027	1.0144
Pathway (6 loci)	AdmixLD	unbalanced	0.0182	0.0305	0.4048	12.6249	0.1591	9.2665
Pathway (6 loci)	Traditional LD	unbalanced	0.6229	0.1313	0.9397	2.4029	0.8955	2.1787
Pathway (6 loci; var. allele. freq.)	AdmixLD	unbalanced	0.0234	0.0384	0.5912	14.7248	0.3814	12.2281
Pathway (6 loci; var. allele. freq.)	Traditional LD	unbalanced	0.4046	0.1878	0.935	2.8153	0.8901	2.631

Supplementary Table S2. True-positive and false-positive rates of the FDR-based outlier detection (FDR < 0.01) across 100 replicates for each simulation scenarios, under balanced (bal) and unbalanced (unbal) sampling. Hit rates are reported across up to five pair categories: True pair — hits between two loci that are genuine DMI or pathway partners; cross pair — hits between two selected loci belonging to different, unrelated DMI pairs (multi-locus DMI scenarios only); background pair — hits among pairs where both markers are >20 kb from any selected locus (the false-positive rate); region pair — hits among pairs where both markers are within 20 kb of a selected locus. Region vs background enrichment is the fold-enrichment of the region pair rate over the background pair rate.

Simulation type	Sampling	Background hit rate (false positives)	Cross pair hit rate	True pair hit rate (true positive)	Region pair hit rate	Region vs background hit rate enrichment
neutral	balanced	0.00008	NA	NA	NA	NA
single DMI (strong)	balanced	0.00047	NA	1	0.4978	1059.15
single DMI (weak)	balanced	0.00009	NA	0.3	0.0114	126.67
multi-DMI (2 loci pairs)	balanced	0.00147	0.97667	1	0.45306	308.2
multi-DMI (3 loci pairs)	balanced	0.003	0.89889	0.92667	0.41512	138.37
multi-DMI (6 loci pairs)	balanced	0.0057	0.10143	0.12	0.05736	10.06
Pathway (6 loci)	balanced	0.0004	NA	0.8925	0.15784	394.6
Pathway (6 loci; var. allele. freq.)	balanced	0.00093	NA	0.99833	0.06381	68.61
neutral	unbalanced	0.00024	NA	NA	NA	NA
single DMI (strong)	unbalanced	0.00059	NA	1	0.45182	765.8
single DMI (weak)	unbalanced	0.00024	NA	0.36	0.01704	71
multi-DMI (2 loci pairs)	unbalanced	0.00159	0.97333	0.975	0.43517	273.69
multi-DMI (3 loci pairs)	unbalanced	0.00296	0.89556	0.9	0.40908	138.2
multi-DMI (6 loci pairs)	unbalanced	0.00051	0.01381	0.025	0.00833	16.33
Pathway (6 loci)	unbalanced	0.00062	NA	0.885	0.15694	253.13
Pathway (6 loci; var. allele. freq.)	unbalanced	0.00103	NA	0.99583	0.05524	53.63

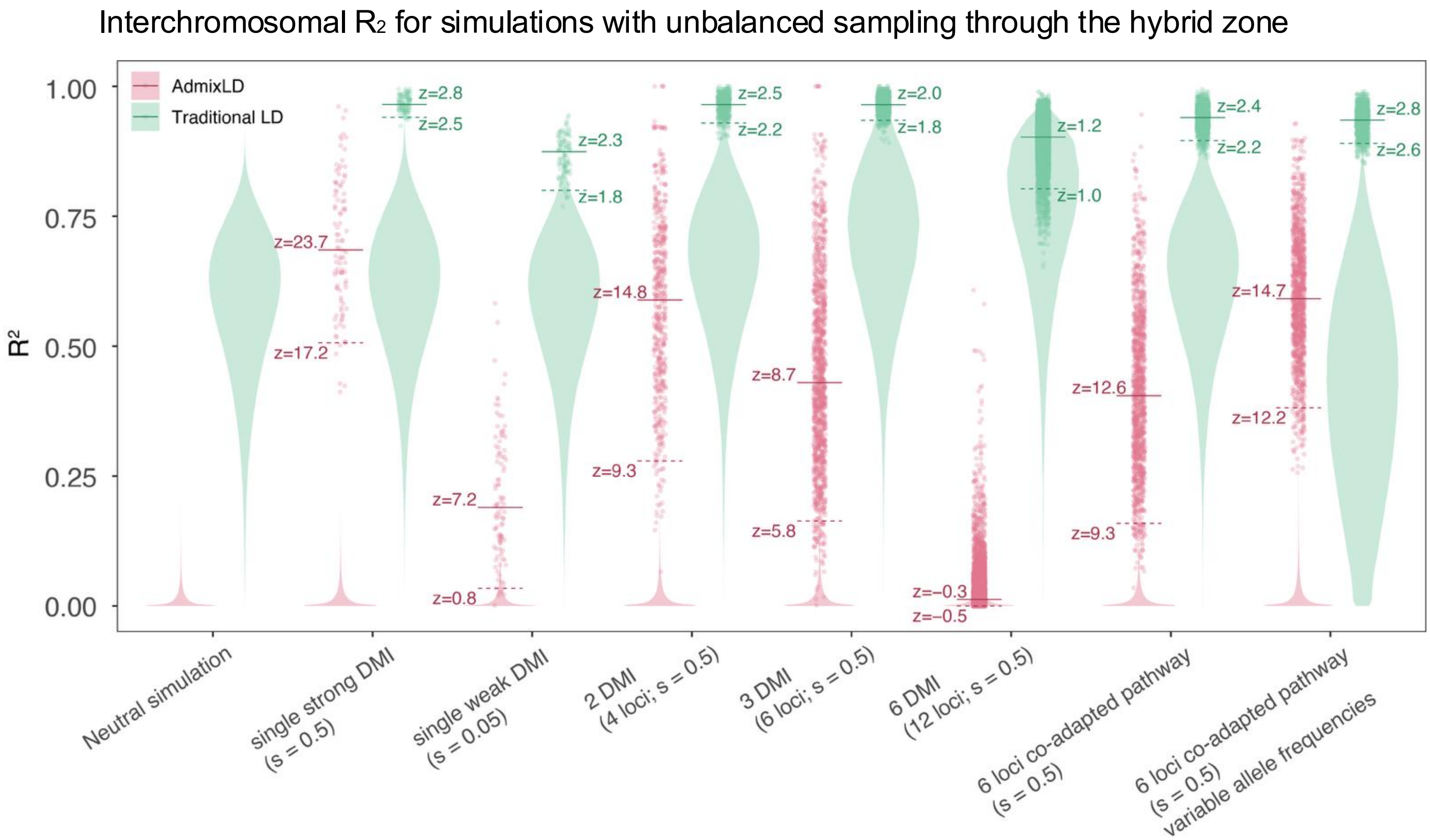

Supplementary Figure 1. Comparison of excess ancestry LD (partial correlation) and traditional LD across different simulation scenarios with unbalanced sampling. Overall R^2 distributions compared to selected loci pairs. Shaded distributions show background R^2 values across all locus pairs; colored points indicate R^2 values for incompatible locus pairs. Pink: excess ancestry LD (AdmixLD); green: traditional LD. Dashed and solid horizontal lines indicate the 5th percentile and median z-scores of incompatible locus pairs, respectively.

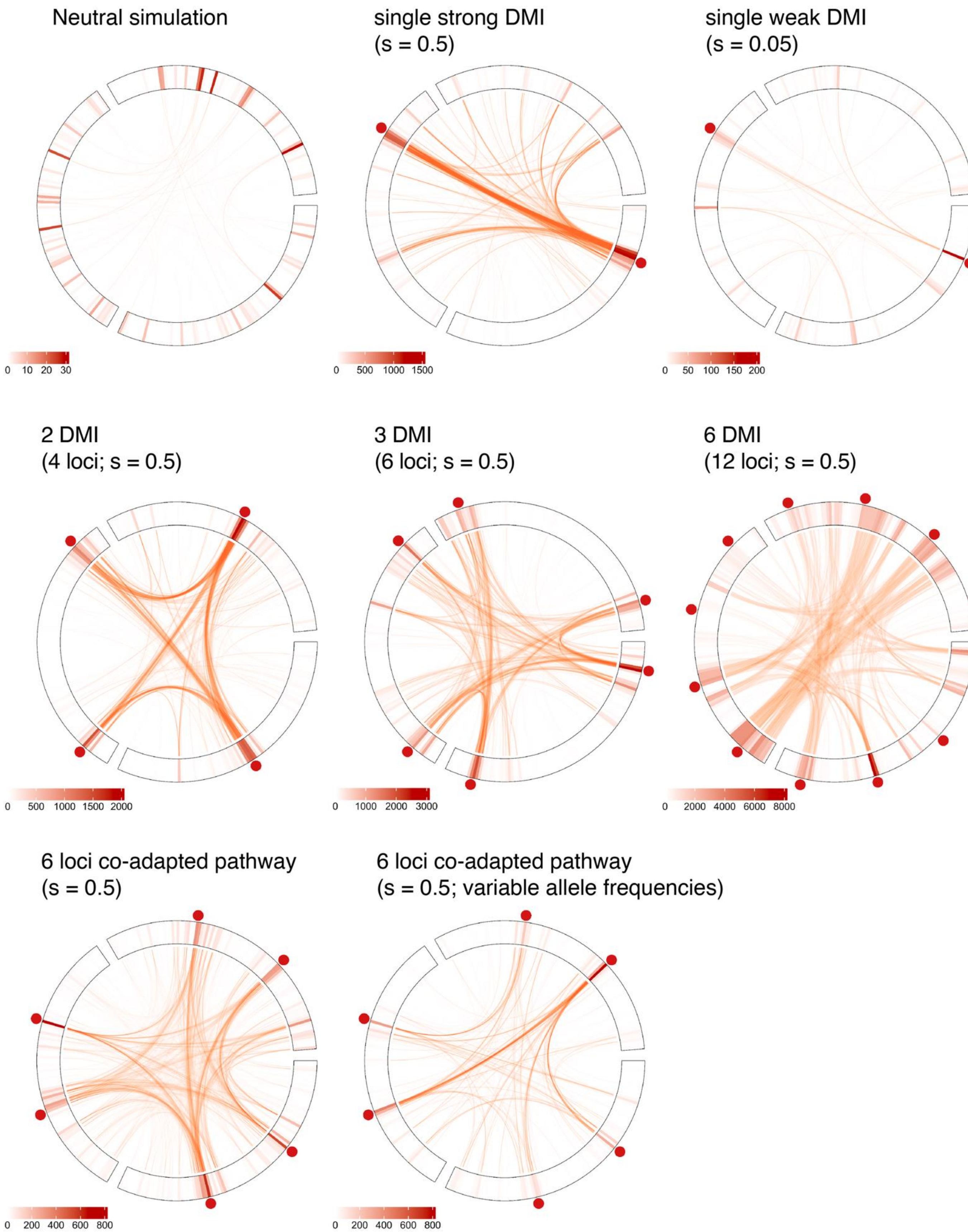

Supplementary Figure 2. Examples of simulation LD hits (FDR < 0.01) with AdmixLD for all simulation scenarios. Structure of identified outlier pairs (using AdmixLD implemented FDR method) across simulated genomes, shown for single simulation replicates. Orange lines represent LD between loci pairs identified as outliers (FDR < 0.01), red dots mark the location of loci under selection, and the outer track show the density of outlier hits.
